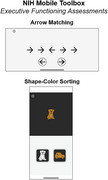# Cross validation of novel secondary executive functioning scores in the Mobile Toolbox for monitoring cognitive change

**DOI:** 10.1002/alz70857_099517

**Published:** 2025-12-24

**Authors:** Y. Catherine Han, Cindy J. Nowinski, Elizabeth M Dworak, Augusta Kamila Zukauskas, Miriam Novack, Sarah Pila, Richard C. Gershon

**Affiliations:** ^1^ Northwestern University Feinberg School of Medicine, Chicago, IL, USA

## Abstract

**Background:**

Executive Function (EF) has multiple components (e.g., attention, inhibitory control, etc.) which can be differentially affected by AD/ADRD. Specific EF deficits can be early indicators of cognitive impairment, providing clues about etiology and likelihood of disease progression. The NIH Mobile Toolbox (MTB) includes adapted, self‐administered measures from the NIH Toolbox® (NIHTB) on a smartphone that are brief, sensitive, and easy‐to‐use for tracking cognitive functioning across the lifespan. The primary scores produced for MTB EF measures (Arrow Matching; Shape‐Color Sorting) are rate‐based scores (# correct/time to complete), which have been shown to be reliable and valid (Novack et al., 2024). In the current study, we expand on these measures by investigating a series of secondary scores (anticipation errors, error rate, and median response time for incorrect and correct responses) towards a more comprehensive EF assessment.

**Method:**

Participants were administered the MTB in person on study‐provided iOS smartphones and completed the NIHTB measures on study‐provided iPads (Study 1: *n* = 92, aged M=49.27, SD=17.65). Participants also completed external measures intended to assess convergent validity (e.g., D‐KEFS, WCST) and divergent validity (e.g., PPVT‐5, NIHTB PV). Another group of participants completed the NIHTB in person and MTB remotely using their own Android/iOS smartphone no more than 14 days after the first session (Study 2: *n* = 1021, aged M=43.97, SD=21.24).

**Result:**

Across studies, there was a significant relationship between all novel scores in both tasks and age (*ρ* = [.27,.60], *p*s < .05); older individuals made more anticipation and total errors and were slower to make correct and incorrect responses. Most scores showed significant, expected correlations against concurrent measures (absolute value *ρ* = [.13,.77], *p*s < .05). Most scores showed non‐significant, low correlations with the proposed divergent measures (*ρ* = [‐.01,.09], *p*s > .05), except for anticipation and total error scores, which showed significant but weaker negative relationships with vocabulary measures. All scores showed strong internal reliability via split‐half correlations (Study 2; SCS median = .94, 25‐75th percentile=[.93,.94]; AM median = .98, 25‐75th percentile=[.97,.98]).

**Conclusion:**

Expanded scores from the MTB EF measures are valid and may be suitable for clinical and pharmaceutical studies, particularly relating to age related cognitive changes. Additional clinical validation is underway, including populations at risk for or diagnosed with cognitive impairment or AD/ADRD.